# Antibiotic and colistin resistance pattern of *Salmonella* spp. isolated from pediatric patients with diarrhea in the Southern region of Vietnam

**DOI:** 10.1016/j.nmni.2025.101576

**Published:** 2025-03-05

**Authors:** Nga Thi Le, Phuong Hoai Hoang, Chinh Van Dang, Tinh Huu Ho, Phuc Le Hoang, Dinh Quang Truong, Ha Thi Thanh Nguyen, Chuong Van Le, Tuom Thi Tinh Truong, Phuong Nhat Tran, Trang Thi Phuong Phan

**Affiliations:** a Center for Bioscience and Biotechnology, University of Science, Ho Chi Minh City, Viet Nam; b Vietnam National University, Ho Chi Minh City, Viet Nam; c Institute of Public Health, Ho Chi Minh City, Viet Nam; d Children's Hospital No.1, Ho Chi Minh City, Viet Nam; e City Children's Hospital, Ho Chi Minh City, Viet Nam; f University of Medicine and Pharmacy, Ho Chi Minh City, Viet Nam; g Laboratory of Molecular Biotechnology, University of Science, Ho Chi Minh City, Viet Nam; h Faculty of Medicine, Van Lang University, Ho Chi Minh City, Viet Nam

**Keywords:** *Salmonella*, Pediatrics, Diarrhea, Antibiotic resistance, *Mcr-1*, IncX4

## Abstract

**Background:**

*Salmonella* spp. is a prominent causative agent of pediatric diarrhea, with recent research emphasizing the rising concern regarding antimicrobial resistance (AMR), particularly involving colistin, a last-resort antibiotic.

**Methods:**

This study involved the collection of 500 stool samples from children with diarrhea from two tertiary pediatric hospitals in Ho Chi Minh City. Conventional methods were used to isolate *Salmonella* spp., and antibiotic resistance profiling against seven antibiotics was conducted using the disk diffusion method. Colistin resistance was determined by the microdilution method. Colistin resistance (*mcr*) genes were detected by PCR assay. A conjugation experiment with a multidrug-resistant *Salmonella* strain and *E. coli* J53 was performed, and the plasmids from this *Salmonella* strain were analyzed using bioinformatics.

**Results:**

The *Salmonella* spp. infection rate was found to be 74/500 (14.8 %). The predominant antibiotic resistance phenotype was ampicillin with 53/74 (71.6 %), followed by trimethoprim-sulfamethoxazole with 23/74 (31.1 %). Resistance rates to ceftazidime, cefotaxime and gentamicin were 14/74 (18.9 %), 13/74 (17.6 %) and 6/74 (8.1 %), respectively. Resistance to imipenem was not observed. Colistin resistance was exhibited in 3/74 (4.1 %) of isolates, all harboring the *mcr-1* gene. The plasmid carrying the *mcr-1* gene could transfer to *E. coli*, which belongs to the IncX4 type and contains IS26.

**Conclusion:**

This study provides valuable insights into the antibiotic resistance profiles of *Salmonella* spp. isolated from pediatric patients, with particular emphasis on colistin resistance.

## Highlights

• First study on colistin-resistant *S. enterica* in Vietnamese children (0–5 years) with diarrhea.
• Identified *mcr-1* plasmid pNCS320 in colistin-resistant *S. enterica* and its conjugation in *E. coli*.
• PNCS320 plasmid in IncX4, containing insertion sequence IS26, was identified.
• Reveals antibiotic resistance in *Salmonella* spp. from children, stressing colistin resistance.

## 1 Introduction

Diarrhea remains a major cause of mortality among children under five, particularly in low - and middle-income countries (LMICs), where it accounts for 9.1 % of deaths globally [1]. Besides Rotavirus and *E. coli*, *Salmonella* spp. is emerging as a significant etiological factor in pediatric diarrhea [2].

*Salmonella* spp. are rod-shaped, Gram-negative bacteria that infect various hosts, with human Non-Typhoidal *Salmonella* (NTS) infections primarily stemming from contact with infected animals or contaminated environments [3]. While salmonellosis treatment typically does not require antibiotics except for invasive cases, antimicrobial resistance (AMR) in *Salmonella* spp. is exacerbating the global AMR crisis [4].

Vietnam, where NTS is associated with up to 15 % of pediatric diarrheal cases [5], is also facing significant AMR challenges due to indiscriminate antimicrobial use in both animal and human health sectors [6]. In Vietnamese pediatric populations, resistance rates to antibiotics such as ampicillin (59 %) and sulfamethoxazole-trimethoprim (36 %) were notably high, while a concerning 37 % resistance rate to chloramphenicol was also recorded [7]. Studies from 2012 to 2020 reported increasing AMR rates among children with diarrhea in southern Vietnam, with multidrug resistance observed in over 53 % of isolated NTS strains [8,9].

Treating multidrug-resistant Gram-negative bacteria often requires last-resort antibiotics like colistin (polymyxin E), which disrupt bacterial cell walls by binding to lipid A [10]. Resistance to colistin is commonly mediated by mobilized colistin resistance (*mcr*) genes, particularly *mcr-1*, which has been detected in *Salmonella* spp. in various food products in Vietnam [[11], [12], [13]]. Although colistin is rarely used for gastrointestinal infections in clinical practice, it remains a critical option for managing carbapenem-resistant infections in tertiary care settings. A recent study reported a colistin usage rate of 73.2 DOT/1000 patient-days in Vietnamese tertiary hospitals, highlighting its role as a last-resort antibiotic [14].

Current treatment guidelines for *Salmonella enterica* infections in Vietnam prioritize third-generation cephalosporins and fluoroquinolones as first-line options, while carbapenems are reserved for severe multidrug-resistant cases [5,7]. Resistance to fluoroquinolones in NTS isolates in Vietnam ranges from 5 % to 10 %, while TMP-SMX resistance rates are reported to reach 36 %, and carbapenem resistance remains rare in *Salmonella* spp., though it has been observed in other *Enterobacteriaceae* such as *E. coli* and *Klebsiella pneumoniae* [7,9].

Despite efforts to reduce colistin use in livestock, it remains widely used in animal farming to promote growth and prevent disease. Approximately 33 % of pig farms have colistin in their feed, and 11 % of marine aquaculture cage farms use it as an antimicrobial agent, further facilitating the spread of resistance from animals to humans through the food chain. Given the detection of colistin-resistant *Salmonella* strains in food products across Vietnam, especially in the Mekong Delta, it is crucial to investigate colistin resistance in human *Salmonella* infections, particularly in children. However, current research on *Salmonella* spp. from pediatric patients in Vietnam is limited. In this investigation, we sought to determine the presence of *mcr-1* in *Salmonella* spp. infecting pediatric patients and uncover the transfer method of *mcr-1* utilizing conjugative plasmids.

## 2 Materials and methods

### 2.1 Study design, enrollment criteria, and sample information

This cross-sectional study, conducted from March to August 2022, involved 500 stool samples, each collected from a unique pediatric patient (aged 0–5 years) with diarrhea at Children's Hospital No.1 and City Children's Hospital in Ho Chi Minh City. **Diarrhea was defined as the passage of loose, watery stools at least three times per day. Patients that were on antibiotics within 6 days prior to admission were excluded.** . Stool samples were collected, preserved, and processed within 24 h according to Vietnam's Ministry of Health guidelines. A structured questionnaire was used to gather demographic and clinical data for each patient.

### 2.2 Isolation and identification of *Salmonella* spp

Stool specimens were inoculated in Selenite Cystine Broth and then incubated on Salmonella Shigella agar at 37 °C for 24 h [15]. Suspected *Salmonella* spp. colonies were further incubated on Nutrient agar and identified using biochemical tests. Colonies that were oxidase-negative and positive for glucose fermentation were preserved in Tryptic Soy Broth with 15 % glycerol at −80 °C. Strain identification was confirmed using a MicroScan WalkAway system.

### 2.3 Antimicrobial susceptibility testing

Antimicrobial susceptibility tests were conducted using the disc diffusion method following CLSI 2022 guidelines [16]. Seven antibiotics were tested on Mueller-Hinton agar, with control strains including *K. pneumonia* ATCC 700603 (positive) and *E. coli* ATCC 25922 (negative).

### 2.4 Detection of colistin-resistant phenotype

The minimum inhibitory concentration (MIC) of colistin against *Salmonella* strains was determined using the dilution method per CLSI standards. Control strains were *E. coli* ATCC 25922 and *Pseudomonas aeruginosa* ATCC 27853, with distilled water as a negative control. Colistin sulfate was diluted in cation-adjusted Mueller-Hinton broth with a 2-fold decreasing concentration from 128 μg/mL. Bacterial suspensions (10^6^ cells/mL) were added to the antibiotic solution in 96-well plates and incubated for 16–20 h at 37 °C. MIC was defined as the lowest antibiotic concentration inhibiting bacterial growth, compared to the CLSI cutoff of 2 μg/mL for *Enterobacterales*.

### 2.5 Colistin resistance genes detection

All isolated *Salmonella* strains were screened for the presence of colistin resistance genes (*mcr-1* to *mcr-5*, *mcr-6* to *mcr-9*) using multiplex PCR. The selection of primers and PCR conditions were performed as described in previous reports for *mcr-1* to *mcr-5* genes [17] and *mcr-6* to *mcr-9* genes [18].

### 2.6 Statistical analysis

R software was used to analyze the data. The qualitative variables were described as frequencies and percentages, while the quantitative variables were summarized as means and standard deviations. Associations between variables were determined using the chi-square test. An association was considered significant when the p-value was <0.05.

### *2.7* *In vitro* plasmid conjugation experiment

To assess the transferability of the colistin resistance gene (*mcr-1*), a conjugation assay was conducted using the donor *Salmonella* strain NCS320 (*mcr-1*-positive) and the recipient *E. coli* J53 (sodium azide-resistant). Cultures of both strains were grown to an OD600 of 0.6 in LB medium at 35 °C. Equal volumes (50 μL) of donor and recipient cells were mixed and plated on MHA agar containing colistin (2 μg/ml) and sodium azide (100 μg/ml). Following overnight incubation at 35 °C, transconjugants were identified and confirmed for *mcr-1* plasmid presence via PCR, and their identity as *E. coli* was verified using a MicroScan WalkAway system [17,19].

### 2.8 Plasmid profile analysis

Plasmids from *Salmonella* spp. NCS320 were extracted using the GeneJET Plasmid Miniprep Kit (Thermo Fisher Scientific). Libraries were prepared with NEBNext® UltraTM II DNA Library Prep Kit for Illumina, and sequencing was performed on a MiniSeq System (Illumina). Sequences were assembled *de novo* from short reads using Unicycler (v0.4.8). The assembled sequences were aligned with a curated plasmid database using BLASTn v2.13.0 [20,21]. Antibiotic resistance genes were identified using ABRIcate v1.0.1 and mcroni v1.0.4 in the CARD database [22]. Insertion Sequence (IS) regions were detected with PANISA v0.1.6, mobileOG-beatrix-1.6, and BLASTN v2.13.0 in the IS-finder database [23,24].

## 3 Results

### 3.1 Clinical and epidemiological features

We first characterized notable features of collected samples (Table 1). Out of the 500 stool samples analyzed, 74 (14.8 %) tested positive for *Salmonella* spp. Some statistically significant findings (p-value <0.05) were observed using univariate statistics: the younger the patient's age, the higher the rate of *Salmonella* spp. infection (20.6 % in the <12-month group, 11 % in the 12–36 months group, and 8.9 % in the 36–60-month group, p-value = 0.008).The rate of *Salmonella* spp. detection was higher in children with blood in stools than in those with non-bloody stools (27.4 % vs. 14.9 %; p = 0.001). These findings highlight the prevalence and clinical impact of *Salmonella* infections in this pediatric population.

**Table 1:** Epidemiological and clinical characteristics of 500 pediatric patients and those infected with ***Salmonella* spp.**

Characteristic		*Salmonella* spp.		p-value
		Yes (n, %)	No (n, %)	
Sex	Male	53 (16.2)	275 (83.8)	0.24
	Female	21 (12.2)	151 (87.8)	
**Age (months)**	<12 months	43 (20.6)	166 (79.4)	**0.008**
	12–36 months	27 (11.0)	219 (89.0)	
	36–60 months	4 (8.9)	41 (91.1)	
**Vomiting**	Yes	10 (11.5)	77 (88.5)	0.34
	No	64 (15.5)	349 (84.5)	
**Blood in stools**	Yes	20 (27.4)	53 (72.6)	**0.001**
	No	54 (14.9)	371 (87.3)	

Table 1 summarizes the epidemiological and clinical variables observed in pediatric patients with *Salmonella* spp.

### 3.2 Antimicrobial and colistin susceptibility

The antimicrobial susceptibility profiles of 74 *Salmonella* spp. isolates are summarized in Table 2 and Table S1. Resistance to AMP was the most prevalent among the isolates, observed in 53 (71.6 %), followed by resistance to third-generation cephalosporins, with resistance to CAZ observed in 14 (18.9 %) and to CTX in 13 (17.6 %). Resistance to CN was observed in 6 (8.1 %), while CIP showed resistance in 4 (5.4 %). SXT resistance was only exhibited in 2 strains, while no resistance was detected for IPM.

**Table 2:** *Salmonella* spp. profiles against various antimicrobials.

Antibiotic	Resistant [n (%)]	Intermediate [n (%)]	Susceptible [n (%)]
Ceftazidime [CAZ] (30 μg)	14 (18.9)	6 (8.1)	54 (73)
Cefotaxime [CTX] (30 μg)	13 (17.6)	17 (23)	44 (59.5)
Ampicillin [AMP] (10 μg)	53 (71.6)	2 (2.7)	19 (25.7)
Imipenem [IPM] (10 μg)	0	3 (4.1)	71 (95.9)
Gentamicin [CN] (10 μg)	6 (8.1)	1 (1.4)	67 (90.5)
Ciprofloxacin [CIP] (5 μg)	4 (5.4)	4 (5.4)	66 (89.2)
Sulfamethoxazole-trimethoprim [SXT] (25 μg)	2 (2.7)	23 (31.1)	49 (66.2)

### 3.3 Detection of colistin-resistant phenotype

Using the MIC determined by the dilution method, it was found that 4.1 % (3/74) of the isolates exhibited resistance to colistin. Subsequent PCR analysis confirmed the presence of *mcr-1* in all the strains, with one strain having a colistin MIC of 4 μg/mL and two strains having a MIC of 8 μg/mL (Table S1).

Examination of the multidrug resistance patterns identified a distinct *Salmonella enterica* strain, S320. The strain exhibited the *mcr-1* gene (MIC = 8.0 μg/mL) and demonstrated multidrug resistance phenotypes, including resistance to penicillin and third-generation cephalosporins. To delve deeper into the genetic basis of the antimicrobial resistance phenotype displayed by this strain, conjugation experiments were conducted and plasmids were extracted for subsequent analysis. The resistance phenotypes observed in this strain are shown in Table S1.

### 3.4 Conjugation experiment

**Fig. 1:**
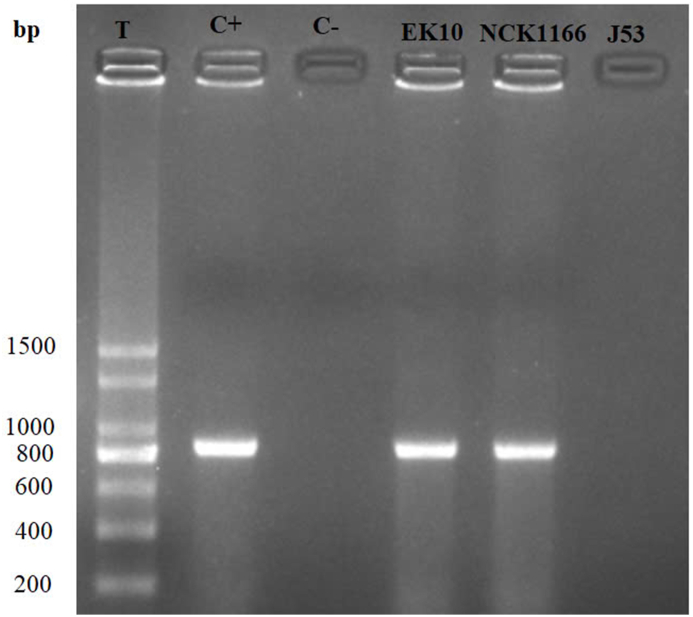
PCR results after conjugation experiment between *Salmonella enterica* S320 and *E. coli* J53 C+: Positive control for *mcr-1* (502 bp); C-: Negative control; S320: *Salmonella* S320 strain; J53: *E. coli* J53 before conjugation; S320+J53: *E. coli* J53 after conjugation.

The strain on the NaN3-colistin plate was identified as *E. coli*, which the PCR results indicated carrying the *mcr-1* gene (502 bp) (Fig. 1). This result demonstrates that *mcr-1* exhibits not only intra-species transferability but also inter-species transferability, extending to bacteria of different species.

### 3.5 Plasmid analysis

Plasmid analysis of the multidrug-resistant *Salmonella* S320 strain, which harbors the *mcr-1* gene, was conducted using the Illumina sequencing method. *De novo* assembly of the sequencing reads resulted in 168 contigs, covering a total length of 5,129,593 base pairs, with the longest contig measuring 474,692 base pairs. During the assembly process, five extrachromosomal elements were circularized, as detailed in Table 3.

**Table 3:** Plasmids originate from *Salmonella* S320.

Name of plasmid	Size (bp)	Plasmid type	Predicted mobility	%GC	Nearest neighbor from NCBI
pNCS320	36441	IncX4	Conjugative	46.9	KY463454
AA374	65445	IncHI1B	Mobilizable	42.7	CP021846
AA152	5687	rep_cluster_2350	Mobilizable	47.1	NC_019136
AB002	89564	rep_cluster_1704	Non-mobilizable	60.6	CP032492
AD312	6215	–	Non-mobilizable	46.1	KX434882

**Fig. 2:**
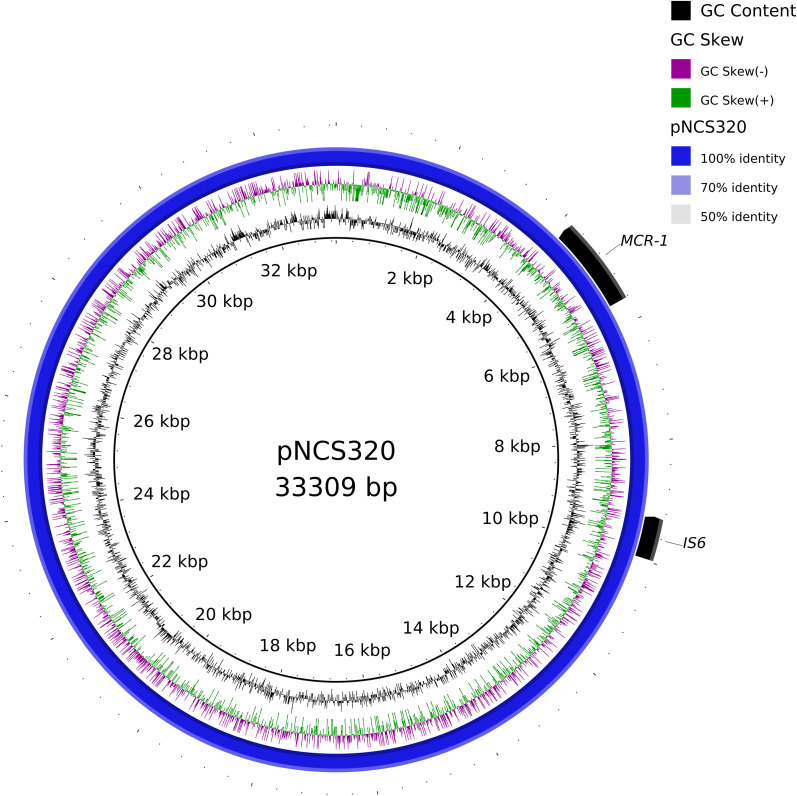
Details of pNCS320 from *Salmonella* S320 strain.

Further analysis using the ABricate tool on the CARD database revealed various resistance genes and IS sequences present on these plasmids (Table 4). The S320 strain carries two significant plasmids associated with resistance genes: pNCS320, an IncX4 plasmid carrying the *mcr-1* gene, and plasmid AA374, an IncHI1B plasmid that harbors *qnrS1*, *blaTEM-1*, and *sul2* resistance genes. Also, both plasmids carried IS sequences of type IS6 and IS2, which could facilitate their ability to transfer resistance genes such as *mcr-1* from *Salmonella* S320 to other hosts. Particularly, the pNCS320 plasmid is of significant interest as it belongs to the IncX4 group and is capable of mobilizing resistance genes (Fig. 2). The plasmid sequence has been deposited in the NCBI database under accession number OR828567.

**Table 4:** Analysis of plasmid sequences.

Name of plasmid	Type	Carried resistance genes	Functions	IS sequences
pNCS320	IncX4	*mcr-1*	Colistin resistance	IS6 (IS26 family)
AA374	IncHI1B	*qnrS1*	Quinolone resistance	IS2 repressor TnpA
		*blaTEM-1*	Beta-lactam resistance	
		*sul2*	Sulfonamides resistance

## 4 Discussion

Based on samples collected from pediatric patients with diarrhea, we found *Salmonella* spp. in nearly 15 % of the samples, highlighting the prevalence and clinical impact of *Salmonella* spp. infections in this population. Younger children show a higher prevalence of *Salmonella* infections, while detection rates of *Salmonella* were also notably higher in children with bloody stools (27.4 %) compared to those without (14.9 %), emphasizing the clinical significance of this symptom and the need for prompt diagnosis and treatment [2]. This study is the first to report the prevalence and horizontal transmission of the *mcr-1* gene in clinical *S. enterica* in Vietnam. The findings indicate that ***mcr-1*** is a key determinant of plasmid-mediated colistin resistance in ***Salmonella* spp.** isolated from pediatric patients hospitalized with diarrhea. In contrast, *mcr-3* is the predominant variant in Thailand [25]. The widespread presence of colistin resistance is likely due to the extensive use of polymyxins in livestock. In Vietnam, *mcr-1* in *Salmonella* spp. has been reported primarily in farming products [[11], [12], [13]]. Horizontal transmission of *mcr-1* from animal to human-associated strains, though rare, could occur via co-existing species like *E. coli* [26].

Gram-negative bacteria are increasingly acquiring resistance through horizontal gene transfer, facilitated by mobile genetic elements. The spread of *mcr-1*-bearing plasmids, such as IncX4, is linked to their high conjugation frequency and the low fitness cost imposed on the host bacterium [27]. Plasmids harboring *mcr-1*, especially IncX4, have been identified in various strains of *Enterobacteriaceae* from clinical, food, and environmental sources across more than 40 countries, suggesting their significant role in the global dissemination of colistin resistance [27,28]. IncX4 plasmids are known to have a strong relationship with colistin usage, when there is a significant reduction in the detection of IncX4 plasmids following a decrease in colistin usage on farms in China [29]. The pNCS320 sequence in this study exhibited pronounced similarity to the plasmid pMCR_WCHEC1618 (accession number KY463454), which corresponds to an *mcr-1*-bearing IncX4 plasmid isolated from *E. coli* in hospital sewage. In a recent report, a plasmid identified in *Klebsiella pneumoniae* isolated from clinical samples also demonstrated a similarity of over 95 % with pMCR_WCHEC1618 [30]. These findings underscore the prevalence of this plasmid in hospital environments and its ability to persist across a diverse spectrum of Gram-negative bacterial strains.

However, this study has some limitations. The use of short-read sequencing may have reduced plasmid assembly accuracy compared to long-read approaches. Additionally, the exclusion of azithromycin and cotrimoxazole from the resistance analysis limits the findings. Future studies should address these aspects to improve the understanding of resistance mechanisms.

In conclusion, our study identified a 14.8 % infection rate of *Salmonella* spp. in pediatric patients with diarrhea in southern Vietnam. Ampicillin resistance was the most prevalent (71.6 %), while no resistance to imipenem was observed. Colistin resistance (4.1 %) was linked to the *mcr-1* gene on an IncX4 plasmid with IS26, capable of transferring to *E. coli*. These findings highlight the critical issue of resistance to last-resort antibiotics like colistin in pediatric *Salmonella* infections.

## CRediT authorship contribution statement

**Nga Thi Le:** Writing – original draft, Supervision, Project administration, Methodology, Investigation, Formal analysis, Data curation, Conceptualization. **Phuong Hoai Hoang:** Writing – review & editing, Writing – original draft, Supervision, Methodology, Formal analysis, Conceptualization. **Chinh Van Dang:** Writing – original draft, Resources, Project administration. **Tinh Huu Ho:** Investigation, Formal analysis. **Phuc Le Hoang:** Writing – review & editing, Writing – original draft, Resources, Investigation. **Dinh Quang Truong:** Supervision, Resources, Investigation. **Ha Thi Thanh Nguyen:** Supervision, Resources, Investigation. **Chuong Van Le:** Writing – review & editing, Writing – original draft, Formal analysis. **Tuom Thi Tinh Truong:** Investigation. **Phuong Nhat Tran:** Writing – original draft. **Trang Thi Phuong Phan:** Writing – review & editing, Project administration, Methodology, Conceptualization.

## Ethical approval

This study was approved by the Ethics Committees of Biomedical Research of Children's Hospital No.1 and City Children's Hospital, Ho Chi Minh City, Vietnam. Consent forms for research participants were signed by the parents or guardians of the enrolled patients.

## Funding sources

This research did not receive any specific grant from funding agencies in the public, commercial, or not-for-profit sectors.

## Declaration of competing interest

The authors declare that they have no known competing financial interests or personal relationships that could have appeared to influence the work reported in this paper.
