# Supplementary material for: Antibiotic and colistin resistance pattern of Salmonella spp. isolated from pediatric patients with diarrhea in the Southern region of Vietnam
Source: New Microbes New Infect. 2025 Mar 5;65:101576. doi: 10.1016/j.nmni.2025.101576 (PMC11937686; doi:10.1016/j.nmni.2025.101576)
Supplement: Multimedia component 1 [file mmc1.docx]

**Table S1**. Antibiotic resistance phenotypes and detection of the ***mcr-1*** gene in ***Salmonella* spp.** strains isolated from stool samples of pediatric patients aged 0-5 with diarrhea.

S: Sensitive, I: Intermediate resistant, R: Resistant

Antibiotic: Ceftazidime [CAZ] (30 µg), Cefotaxime [CTX] (30 µg), Ampicillin [AMP] (10 µg), Imipenem [IPM] (10 µg), Gentamicin [CN] (10 µg), Ciprofloxacin [CIP] (5 µg), Sulfamethoxazole-trimethoprim [SXT] (25 µg).

| **Sample ID** | **CAZ** | **CTX** | **AMP** | **IPM** | **CIP** | **SXT** | **CN** | **ESBL** | **MIC** | ***mcr-1*** |
| --- | --- | --- | --- | --- | --- | --- | --- | --- | --- | --- |
| 11 | S | S | R | S | S | S | S | - | 0 | - |
| 13 | S | S | S | S | S | S | S | - | 0 | - |
| 26 | S | S | R | S | S | I | S | - | 2 | - |
| 32 | S | S | R | S | S | R | S | - | 0 | - |
| 37 | R | R | R | S | I | R | R | ESBL | 0 | - |
| 40 | I | I | R | S | S | S | S | - | 2 | - |
| 54 | S | I | R | S | S | R | S | - | 0 | - |
| 62 | R | R | R | S | S | R | S | ESBL | 0 | - |
| 70 | R | R | R | S | S | S | S | ESBL | 0 | - |
| 71 | S | S | R | S | S | R | S | - | 0 | - |
| 76 | S | S | R | S | S | R | S | - | 0 | - |
| 85 | S | S | R | S | S | S | S | - | 0 | - |
| 93 | R | R | R | S | S | S | S | ESBL | 0 | - |
| 97 | I | I | R | S | S | S | S | - | 0 | - |
| 106 | I | I | R | S | I | R | S | - | 2 | - |
| 108 | S | I | R | S | S | S | S | - | 0 | - |
| 110 | S | S | S | S | S | S | S | - | 0 | - |
| 116 | S | S | S | S | S | S | S | - | 0 | - |
| 125 | S | S | R | I | S | R | S | - | 0 | - |
| 145 | S | S | S | S | S | S | S | - | 0 | - |
| 151 | R | I | S | S | S | S | S | - | 0 | - |
| 154 | S | S | R | S | S | S | S | - | 2 | - |
| 156 | S | S | S | S | S | S | S | - | 0 | - |
| 174 | S | I | R | S | S | S | S | - | 0 | - |
| 181 | I | I | R | S | S | S | S | - | 0 | - |
| 203 | S | S | R | S | S | R | S | - | 0 | - |
| 205 | S | S | R | S | S | S | S | - | 0 | - |
| 213 | S | S | R | S | S | R | S | - | 0 | - |
| 220 | I | I | S | S | S | S | S | - | 0 | - |
| 243 | S | S | R | S | S | S | S | - | 0 | - |
| 260 | S | S | R | S | R | R | S | - | 0 | - |
| 275 | S | S | S | S | S | S | S | - | 0 | - |
| 276 | S | S | S | S | S | S | S | - | 0 | - |
| 278 | S | S | S | S | S | S | S | - | 0 | - |
| 298 | S | S | S | S | S | S | S | - | 0 | - |
| 310 | S | S | S | S | S | S | S | - | 4 | *mcr-1* |
| **320** | R | R | R | S | I | S | S | ESBL | 8 | *mcr-1* |
| 328 | S | I | R | S | S | S | S | - | 0 | - |
| 331 | S | S | R | S | S | I | S | - | 8 | *mcr-1* |
| 345 | I | I | R | S | R | R | S | *-* | 2 | - |
| 362 | S | I | R | I | S | S | S | - | 0 | - |
| 369 | S | S | S | S | S | S | S | - | 0 | - |
| 370 | S | S | R | S | S | S | S | - | 0 | - |
| 374 | S | S | R | S | S | S | S | *-* | 2 | - |
| 403 | S | I | R | S | S | S | S | - | 0 | - |
| 418 | S | S | S | S | S | S | S | - | 0 | - |
| 420 | S | S | S | S | S | S | S | - | 2 | - |
| 434 | S | S | R | S | S | S | S | - | 0 | - |
| 438 | S | I | R | S | S | R | S | - | 0 | - |
| 441 | S | I | R | S | S | R | S | - | 0 | - |
| 457 | S | S | R | S | S | S | S | - | 0 | - |
| 461 | S | S | R | S | S | R | R | - | 0 | - |
| 462 | S | S | R | S | S | S | S | - | 0 | - |
| 463 | S | S | R | S | S | S | S | - | 0 | - |
| 465 | S | S | R | S | S | R | S | - | 0 | - |
| 472 | S | I | I | S | S | S | S | - | 2 | *-* |
| 474 | R | R | I | S | R | R | R | ESBL | 2 | *-* |
| 495 | R | R | R | S | R | R | I | ESBL | 0 | *-* |
| 512 | S | S | S | S | S | S | S | - | 0 | *-* |
| 518 | R | R | R | S | S | R | R | ESBL | 0 | *-* |
| 531 | S | S | R | S | S | R | S | - | 0 | *-* |
| 538 | S | S | S | S | S | S | S | - | 0 | *-* |
| 560 | R | R | R | S | I | R | S | ESBL | 0 | *-* |
| 572 | S | S | S | S | S | S | S | - | 0 | *-* |
| 597 | S | I | S | S | S | S | S | - | 0 | *-* |
| 604 | R | R | R | S | S | R | S | - | 0 | *-* |
| 614 | R | R | R | S | S | R | S | ESBL | 0 | *-* |
| 647 | R | R | R | S | S | S | R | ESBL | 0 | *-* |
| 651 | R | R | R | S | S | S | S | ESBL | 0 | *-* |
| 669 | S | S | R | S | S | S | R | - | 0 | *-* |
| 674 | S | S | R | S | S | S | S | - | 0 | *-* |
| 677 | S | S | R | S | S | S | S | - | 0 | *-* |
| 686 | S | S | R | S | S | S | S | - | 0 | *-* |
| 688 | S | S | R | I | S | S | S | - | 0 | *-* |
